# Public Service Motivation and Determining Factors to Attract and Retain Health Professionals in the Public Sector: A Systematic Review

**DOI:** 10.3390/bs12040095

**Published:** 2022-03-29

**Authors:** Alexandre Fernandes, Gonçalo Santinha, Teresa Forte

**Affiliations:** Governance, Competitiveness and Public Policies (GOVCOPP), Department of Social, Political and Territorial Sciences, University of Aveiro, 3810-193 Aveiro, Portugal; g.santinha@ua.pt (G.S.); teresaforte@ua.pt (T.F.)

**Keywords:** health professionals, public sector, public service motivation, systematic literature review

## Abstract

(1) Background: The motivational determinants of health professionals to choose and remain in the public sector have been increasingly addressed, including the customized approach of Public Service Motivation (PSM). However, to date, no systematic research overview has been performed in this domain, leaving the body of literature unstructured. This article fills this gap by assessing the motivational factors of choice for the public sector in the health field, and the conceptual and methodological trends of this research stream. (2) Methods: This study follows the PRISMA protocol to ascertain patterns in past research and inform researchers, practitioners, and policymakers. Eighty-nine documents published between 1998 and 2021 were retained after selecting them according to their theme and outlined goals. (3) Results: Common motivational determinants are remuneration, available resources, work conditions, and frequency of contact and interaction with patients. The PSM construct and scale are often employed as main frameworks, but there is also a concern in assessing motivation drawing on psychological constructs that reflect the challenging line of work and environment that is health care, such as presenteeism, stress, and perception of hindrances. (4) Conclusions: By focusing on health professionals’ motivation, this study contributes to a timely systematization in challenging times for health institutions and their human resources.

## 1. Introduction

Public Service Motivation (PSM), as conceptualized by Perry and Wise (1990) [1], encompasses individual beliefs, motivations, values, and attitudes towards the nature and mission of public institutions. The interest in PSM as a determining factor for choosing and staying in public administration gained traction in recent years [2,3,4], shifting the way motivation was addressed in light of New Public Management (NPM). Mimicking the private sector, the NPM showed concern for efficiency, efficacy, productivity, and performance, which are of growing importance for decision makers, researchers, and professionals [5,6,7], but with counterproductive consequences in the recruitment of personnel upholding the desired public values. In fact, the use of the private sector managerial style in public administration has had a critical impact in the human resources domain, namely in the loss of specific features usually regarded as attractive in human resources, such as the guarantee of job security and more predictable promotion opportunities [8].

Although public sector workforce recruitment has been systematically addressed in the literature as a problematic issue that needs to be tackled [8], even if there is no consensus on the measures and practices to promote positive change [9], employees’ motivation to choose and remain in the public sector, however, has been overlooked for a long time, generating a gap in understanding how to recruit professionals aligned with public values.

The constructs of Public Sector Motivation and Public Service Motivation contributed prominently to this topic, by tailoring an approach to motivation adapted to features of the public sector. Public Sector Motivation emphasizes acting in one’s self-interest and maximizing individual choice, positive outcomes, and extrinsic rewards, such as job security, job progression, and salary [8,10]. Public Service Motivation (PSM) (Perry and Wise, 1990) [1] focuses on the entanglement between individual beliefs, motivations, values, and attitudes, and the nature and mission of public institutions [1,11]. PSM’s original measurement includes four conceptual dimensions: attraction to the public interest, participation in the processes of political decisions, compassion, and self-sacrifice [12], with a transcultural adaptation with 16 items [13]. This last construct has shown to influence organizational behavior [14], the choice of a sector of activity, performance [15], and workers’ satisfaction [16]. Primarily, PSM was circumscribed for public institutions and organizations, then being argued further on that its essence is also found outside these boundaries [17].

Within this context, debates have emerged around the concepts of intrinsic and extrinsic motivation [18]. This distinction proves to be useful in framing the choice determinants of the public vs. private sector. For instance, it is argued that public sector employees are more concerned with work safety and less with remuneration as opposed to employees from the private sector, who tend to aim for high paychecks [19].

Health professionals are the backbone of any health system, and their motivation and behavior significantly influence the performance of a health system [20]. Moreover, universal health coverage cannot be achieved without a workforce aligned with that purpose and practice [21,22]. Still, professionals in the health field are recognized by the World Health Organization as representing the main cost and investment in health care provision, due to job requirements, the financial and non-financial investments, and the continuous investment in education to maintain the quality of their work [23].

To this adds the current context of COVID-19, as it is particularly demanding for health professionals to stay focused, motivated, and feel safe facing such a challenge. The enlargement and allocation of the health workforce demand more strategic approaches regarding the number, availability, and distribution of health professionals; the training of specific competences and skills to deal with COVID-19; the guarantee of safe and decent work conditions; fast enactment of policies and regulations; and support mechanisms, including financial resources [24].

The growing importance of the theme of motivation in the past decade underlines the relevance of aggregating the studies conducted to draw conclusions for future research or interventions. No similar review has been conducted comparing several world regions with a special focus on the developing world [25]. In this sense, the main goals of this work are:

(i) To characterize the research about health professionals’ motivational determinants, including the methodological quality, geographical distribution, choice of journals, cross-country and author collaboration, and co-citation trends;
(ii) To identify the motivational factors influencing the sector’s choice by health professionals in different territories;
(iii) To identify the dissemination and use of the main instruments to measure PSM; and
(iv) To identify the main motivational theories supporting the studies.

The remaining sections outline the description of the methods used, including the review protocol and the search process, followed by the results and a discussion of the obtained findings, ending with some concluding remarks and suggestions/limitations for future research.

## 2. Materials and Methods

### 2.1. Research Questions

Systematic reviews of literature contribute to providing an updated state of the art of a given topic and clarify future research avenues [25]. This is achievable by highlighting important questions and gaps in the literature [26,27].

This review employed the PRISMA (Preferred Reporting Items for Systematic Reviews and Meta-Analyses) protocol, developed within the context of health sciences to enhance the reliability and replication of literature reviews and meta-analyses [28,29,30]. The selection of studies to address the abovementioned goals answered three research questions:

- What characterizes the research on health professionals’ motivation regarding geographical and authorship distribution, collaborations, and co-citations?
- What are the factors motivating health professionals to choose the public sector for their activity?
- What are the methods and instruments employed to assess health professionals’ motivation?

### 2.2. Search Strategy, Inclusion and Exclusion Criteria

The articles were sourced from three database—SCOPUS, Web of Science (WoS), and PubMed—including studies in the field of social and medical sciences. The expressions searched in article titles, abstracts, and keywords were: [“public service” OR “public sector” OR “public work” OR “public employ*”] AND [“motivation” OR “altruism” OR “ethic*” OR “prosocial”] AND [“health service*” OR “health provider*” OR healthcare OR “health care” OR “primary care” OR hospital* OR nurs* OR doctor* OR “health professional*” OR physician* OR medic* OR “health worker*”]. The search resulted in 2424 documents.

The search was refined by language (English) and type of document according to the database: (i) for SCOPUS, we chose “article” + “conference paper” + “book chapter” + “short survey”; (ii) for WoS, we chose “article” + “other” + “early access” + “unspecified”; (iii) and for PUBMED, all were considered. No time period was outlined in order to analyze the literature evolution according to time. After these stages, duplicated articles were eliminated.

In order to prevent the inclusion of articles not related to the theme, specifically the concept of motivation for public service and/or motivation related to the field of health and health professionals, the final selection of articles comprised the reading of titles and abstracts. The excluded articles focused on fields such as disease and comorbidity, pharmacology, microbiology, and biochemistry. This phase was conducted by one author and further verified by two authors to strengthen the assessment, substantially reducing the number of articles and avoiding the inclusion of non-related articles and the exclusion of relevant articles.

**Figure 1:**
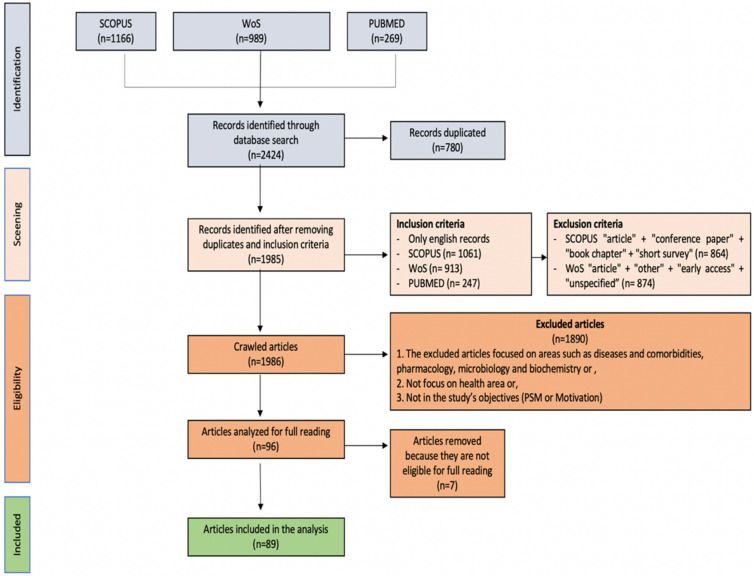
PRISMA flowchart screening processes for narrative synthesis.

Initially, 2424 articles were retained from Scopus (n = 1166), WoS (n = 989), and PubMed (n = 269). Seventy articles were repeated and were thus excluded. Eighty-nine documents were retained after selecting them according to their theme and outlined goals, as shown in the PRISMA diagram below (Figure 1).

Bibliometrix, VOSviewer, and PlumXmetrics were used to process the bibliometric data and the Mendeley bibliographic referencing software to organize the references.

### 2.3. Methodological Quality Assessment

The methodological quality of each of the 89 studies was assessed using a checklist based on the works developed by Pitchforth et al. (2017) [31], Yang et al. (2020) [32], the Critical Appraisal Skills Programme (2013) [33], and Health Evidence (2018) [25]. A component approach, as advocated by the PRISMA statement, was used when applying the checklist, assessing each item individually rather than generating a summary score [34]. The checklist assesses 6 features of the study (Table 1), and responses are scored “yes” or “no”. Two authors independently conducted the quality assessment of each study. Disagreements were resolved by discussion, with the involvement of the third author, and a final decision was achieved by consensus.

## 3. Results

### 3.1. Authors

The 89 articles were published between 1998 and 2021 and involved 297 different authors. The mean value is three authors per article and only 14 articles were written by just one author (15.7%). Author’s affiliations are distributed among several research institutions worldwide, specialized in health management, health policies, and human resources and political sciences. Table 2 presents the authors who published the greatest number of articles within the scope of this study.

The scientific collaboration between authors or institutions shows a solid internationalization of new knowledge. The collaboration index or the average number of authors on multi-authored papers is 3.79; thus, on average, a non-single author publication in this sample has almost four authors.

### 3.2. Publications

The analyzed sample is composed of 88 articles published in 52 scientific journals and one conference paper. Among these, seven journals published three or more articles, namely, *Human Resources for Health, Health Policy and Planning, BMC Health Services Research, International Journal of Public Administration, Global Health Action, International Public Management Journal*, and *Social Science and Medicine*. Most of these are first-quartile (Q1) journals under the scope of health policies and/or public administration (Table 3).

**Figure 2:**
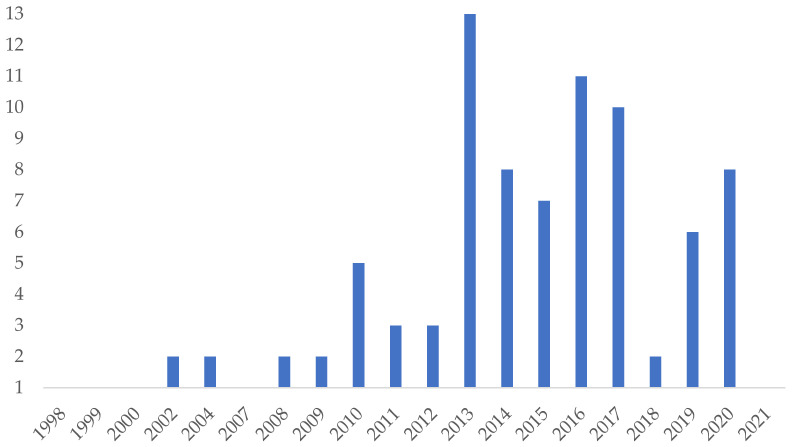
Number of publications per year.

The topic under analysis is quite recent, particularly in the health field, but steadily growing, with an increase of 12.25% of articles per year. As shown in Figure 2, the majority of the publications are from 2013 (n = 13), increasing afterwards with two-thirds of the total of publications (n = 66).

### 3.3. Countries

**Figure 3:**
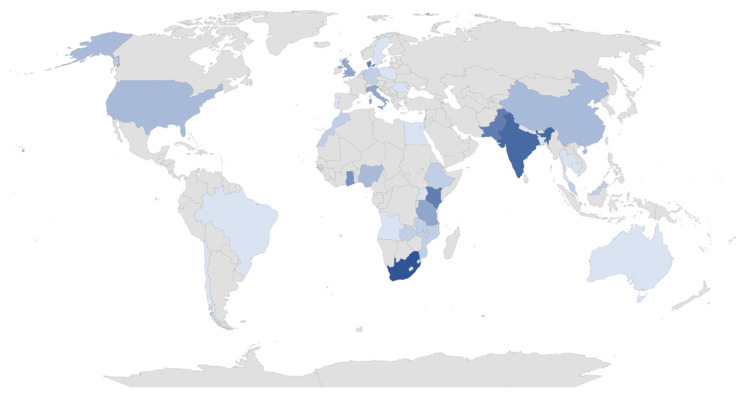
Map with scientific dissemination per country (“Bibliometrix”).

In our sample, the geographical distribution of the articles—based on the contexts of the study—suggest that the interest of PSM when applied to the health field shows a wide geographical dispersion (Table 4 and Figure 3). Interestingly, most of the studies are from non-WEIRD (White, Educated, Industrialized, Rich, and Democratic) samples, particularly African (40%, n = 36) and Asian (26.7%, n = 24) countries. European contexts are the least addressed (24.4%, n = 22). India (7.8%, n = 7), Pakistan (6.74%, n = 6), and Denmark (6.74%, n = 6) are the countries with the most publications on the topic.

The geographical analysis also shows a prevalence of Commonwealth countries. The interest shown may be due to human capital, i.e., the chronic lack of human resources in the health sector in some lesser-developed countries, particularly in Western Africa [13]. In addition, historically, many health professionals of the U.K. emigrated to English-speaking countries, such as South Africa, Pakistan, India, and Nigeria [35]. In Sub-Saharan African countries, and particularly in rural and remote areas, there are few human resources, driven by the often poor working conditions in those contexts [36]. On the other hand, Danish workers were considered the most satisfied in their workplace and can serve as an example for the health system/context and conditions in which they operate [37]. This being said, the distribution of countries involved in the research prevents us from generalizing and providing exact reasons.

### 3.4. Analysis of Scientific Collaboration

**Figure 4:**
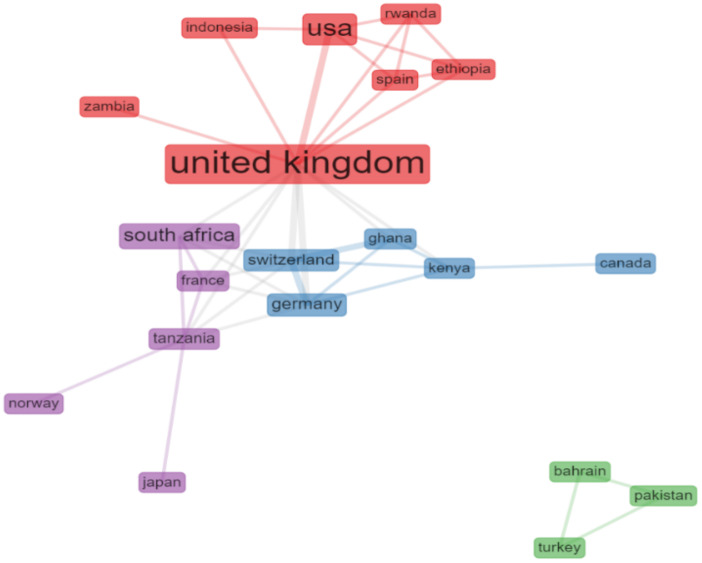
Collaboration between countries (“Bibliometrix”).

The more significant interactions between countries (Figure 4 and Table 5) and authors (Figure 4) are presented below, emphasizing links that represent co-authorships [38].

#### 3.4.1. Cross-Country Collaboration

Collaboration between countries in these studies has its main central node in the U.K. (betweenness centrality = 71.083), followed by the USA, Germany, and Switzerland. These results corroborate the salience of the scientific field in Anglo-Saxon countries. Table 4 depicts the publications based on the main ten countries. Most are published within one country; among these, the U.K. and the USA present more publications and collaborations, with an MCP publication rate of 25%.

#### 3.4.2. Collaboration among Authors

**Figure 5:**
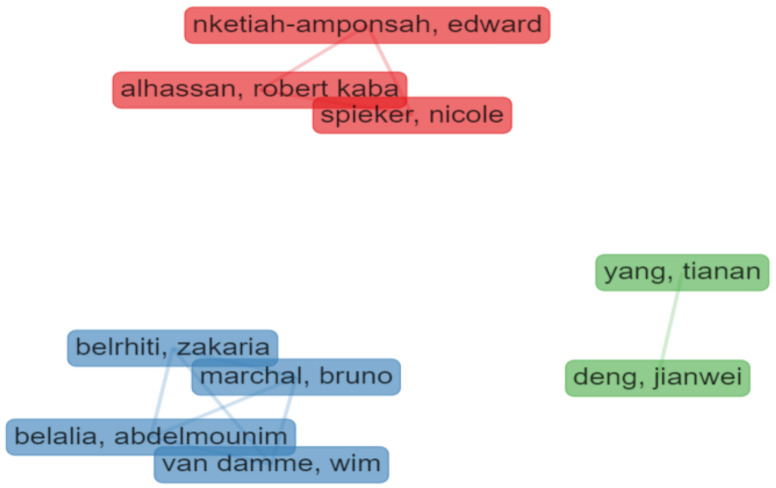
Collaboration among authors (“Bibliometrix”).

In the analysis of the authors’ collaborations, only one-shot collaborations were excluded (min. edge = 2), resulting in nine authors identified in three clusters (Figure 5):

- Cluster 1 (in blue) includes four authors from Morocco and Belgium and focuses on motivation and public sector choice;
- Cluster 2 (in red) includes three authors from The Netherlands and Ghana and mostly pays attention to the quality of health services and human resources; and
- Cluster 3 (in green) is based on two Chinese authors from the Institute of Technology in Beijing, focused on health policies management.

#### 3.4.3. Co-Citation Analysis

The co-citation analysis, representing the frequency of which two documents are cited together in the literature, thus being conceptually close to each other [39], is depicted in Figure 5. The items are represented by a label, pattern, and circle, the size of which is determined by the weight it has in the network, connected by the links (lines). Usually, the closer the authors, the stronger their co-citation link, which is also represented by the lines.

**Figure 6:**
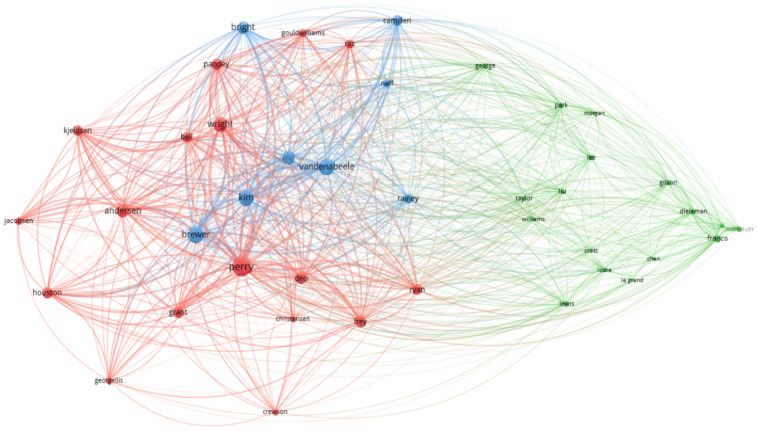
Analysis of co-citations—authors.

Their graphic representation is similar to the net visualization with three clusters, also represented by blue, green, and red (Figure 6). In Figure 6 and Table 6, the larger number of items corresponds to the more yellow and dense nodes. Three clusters (blue, red, and green) represent the data using the authors’ surnames. The interpretation of these data is described in Table 6.

**Figure 7:**
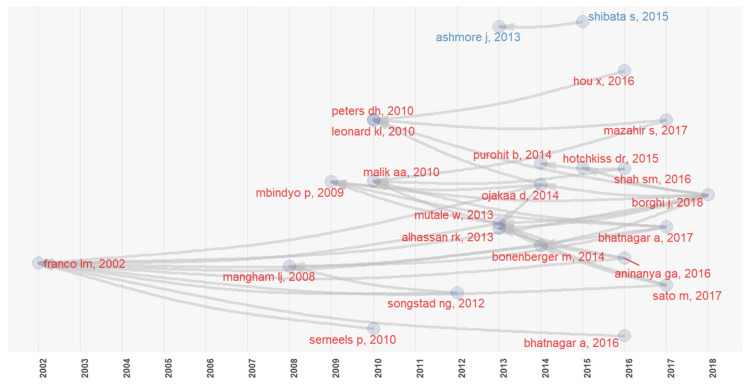
Historiographic analysis (“Bibliometrix”).

The historiographic analysis allows us to identify the research on a given topic while providing a graphic and genealogic presentation of the citation connections [40]. For each article, there is a Global Citation Score (GCS) as well as a Local Citation Score (LCS), which identifies the number of times the article is cited [39]. As shown in Figure 7, Franco et al. (2002) is the most referenced work (LCS = 10/GCS = 363), addressing the determinants of work motivation and the ways in which health sector reform may positively affect workers’ motivation. It is followed by Mutale et al. (2013) [41] (LCS = 5/GCS = 42), who evaluate the motivation shown by rural health workers, and Mbindyo et al. (2009) (LCS = 4/GCS = 81), who explore contextual influences on workers’ motivation and recommend a change in clinical practices in Kenyan hospitals.

### 3.5. Methodological Quality Assessment

**Figure 8:**
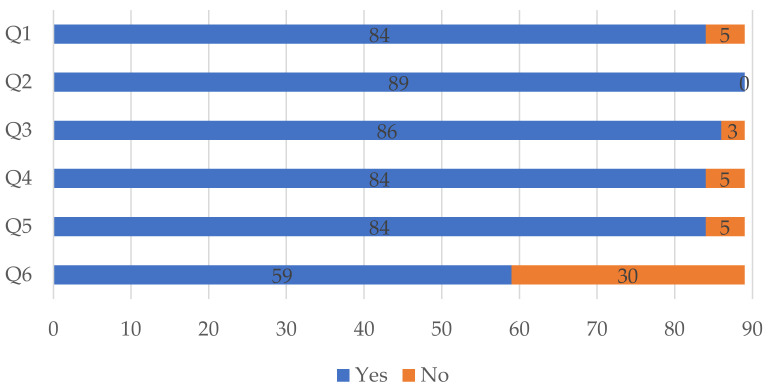
Results of the quality assessment.

Overall, studies performed well according to the quality assessment checklist, as depicted graphically in Figure 8. Eighty-four studies clearly stated the aims of the conducted research (Q1), provided a clear and comprehensive description of the instruments and/or scales used (Q4), and presented a clear statement of the findings (Q5). All eighty-nine studies stated plainly the context of the study (Q2) and only three studies did not explicitly explain and use methods that supported the research in a clear manner (Q3). In turn, thirty studies did not discuss openly and extensively the limitations of their research (Q6).

### 3.6. Object of Analysis

The majority of the studies (n = 56, 62.9%) analyze the public sector exclusively (62.9%, n = 56), while 34.8% (n = 31) compare public professionals with the private sector and only two focus on the private sector.

As for the nature of care, 34.8% (n = 31) are based on an analysis of professionals working at different care facilities, followed by an exclusive focus on hospital workforces (32.6%, n = 29). Some 13.48% (n = 12) studied primary care facilities and 12.4% (n = 11) address college students of health-related degrees (Table 7). The type of professionals in the institutions and in the articles of the study are presented in the Table 8.

### 3.7. Main Motivation Theories

The main motivation theories are addressed in 16 articles (Table 9). Four articles focus on Maslow’s Hierarchy of Needs (Maslow, 1943) [42] (n = 4) and the other theories—Self-Determination Theory (Deci and Ryan, 1985) [43], McClelland Theory (McClelland, 1960) [44], Herzberg Theory (Herzberg, 1959) [45], and the Job Characteristics Model (Hackman and Oldham, 1975) [46]—are addressed by three articles each. Table 10 briefly describes each one of these motivations’ theories.

As shown in Table 9, most of these theories are needs-based theories of motivation, attempting to identify the drives and needs that motivate workers. These theories consider that motivated behavior is driven by individuals’ efforts to satisfy specific intrinsic needs. In turn, the Job Characteristics Model is the only one focusing more on the nature of the task.

It is curious, though, that in our sample, only a few studies are anchored in major theories of motivation. The lack of more theoretically cohesive research may result from the topic being addressed by several research areas, some of them intending to approach the topic more superficially or only in a descriptive fashion. It is also suggestive of a gap in health professionals’ motivation research regarding an integrated conceptual and methodological model tailored to this specific population group.

The main theories of motivation used attempt to identify content and processes that determine motivated behavior. Despite sometimes being referred to as theory (as in five of the articles analyzed), PSM is rather a construct that describes prosocial motivation to serve the public interest and help others. PSM antecedents are seen as focusing on five main factors that may also be shared by the other theories, namely, care, fairness, loyalty, authority, and sanctity [47].

### 3.8. Methods

Almost half of the studies (47.2%, n = 42) are exclusively quantitative, based on surveys, but a significant number are of a qualitative nature, namely interviews (24.7%, n = 22). These preferred instruments are combined solely with focus groups in 6.7% (n = 6) and altogether in three studies (Table 11).

### 3.9. Instruments Used to Measure Motivation and Related Constructs

The first assessment of the public service motivation scale was developed by Perry (1996) [12]. In our sample, only seven of the studies used this four-dimension scale, which was the first to measure this construct through four distinct dimensions: compassion, self-sacrifice, commitment to public interest, and attraction to public policy. It was later subjected to a transcultural adaptation (Kim et al., 2013) [13], involving 12 countries—Australia, Belgium, China, Denmark, France, Italy, South Korea, Lithuania, The Netherlands, Switzerland, the United Kingdom, and the United States—and the four dimensions were updated to attraction to public service, commitment to public values, compassion, and self-sacrifice.

Particularly, in the quantitative studies, there is a wide dispersion of the type of instruments used, addressing related constructs that impact the motivation of health professionals, including inflexible work schedules, long hours, and heavy workloads, which are known to cause high levels of stress and impair their motivation and efficiency. Thus, many of the scales employed do not measure motivation per se (as the PSM scale) but other factors that impact motivation at work. An example is the Challenge and Hindrance-Related Self-Reported Stress (C-HSS), with 11 items, six measuring stress-related challenges and five using stress-related obstacles. Developed by Cavanaugh (2000) [48] (n = 4), C-HSS addresses stress in relation to organizational responsibilities, position, insecurity, and resources. The Presenteeism Scale [49] also employed, in turn, addresses important processual and behavioral aspects of how health professionals feel at work, namely, the level of stress awareness, the pleasure in performing tasks, and the energy and concentration to conclude tasks, all important intrinsic motivators. On the other hand, the Job Satisfaction Scale [50] and Job Performance Scale [51] are also focused on important constructs that determine motivation, namely, satisfaction and performance evaluation, more in line with the process theories of motivation regarding behavior that attempt to identify the variables that may go into motivation. The assessment of related variables, and not the determinants of motivation themselves, may also explain the lack of more studies with a strong theoretical basis.

Forty-four studies employ instruments validated by other authors and 45 use instruments created by their own authors based on (i) the motivational constructs for public service and (ii) the intrinsic and extrinsic factors that health professionals value in the public or private sector. This profusion of adaptations, especially considering the wide geographical dispersion of the studies, impairs the further fine-tuning of major measurement tools as well as sound systematic comparisons between the same constructs.

### 3.10. Main Extrinsic and Intrinsic Factors

The main motivational factors mentioned by health professionals in the analyzed studies (see Table 12) are those associated with a concern at a pre-entry level in the public sector and present, overall, a clear negative valence, namely, low salaries (n = 47), few material resources and work conditions (n = 23), and frozen careers (n = 11). Only three cases are related to satisfaction with salary, career progression, and favorable work conditions, and only in seven cases is a suitable work environment reported, comprising general factors such as appropriate work conditions, financial incentives and bonuses, and professional recognition and respect from supervisors and patients.

As for the main intrinsic motivational factors, reports on absenteeism are very common (n = 22) (see Table 13). Absenteeism is the recurrent absence of the worker during the normal schedule of daily work, a common reality in countries in Sub-Saharan Africa [52], which may be related to the location of health units in rural contexts with longer commuting, unpleasant and poor work environments, among other factors.

However, overall, studies refer to other elements with a positive valence, namely, contact with patients (n = 21), work safety (n = 16), and the recognition of their work (n = 11).

## 4. Discussion of Intrinsic and Extrinsic Factors

### 4.1. Work Environment

The studies analyzed reveal that the lack of communication between coworkers in the workplace, the nature of the supervision, the personal values of the professionals, the low salaries, and the work conditions affect motivation and may promote an undesirable work environment (e.g., [46,47,48,49,50,51,52,53,54,55,56,57,58,59] (see Appendix A)).

A weak politics of recruitment may cause uneasiness [60]. As shown in article [50], there is a political interference in public sector hospitals’ management and, as such, a perceived better work environment in the private health sector. On the other hand, notwithstanding the better extrinsic work conditions in the private sector, article [61] postulates that health professionals of the NHS (British health system) do not necessarily exclude the public sector in their pursuit of a suitable work environment. Article [59] identifies inland areas as more prone to stressful work environments, particularly when basic conditions such as housing and comfortable commuting are lacking.

### 4.2. Career Progression

Eight studies [60,62,63,64,65,66,67,68,69] highlight that delays in career progressions are substantial demotivators, corroborating the motivational power of this particular reward. Regardless of external determinants (e.g., financial crisis or hospital management), to perceive chances of being promoted is an important motivator. The contrary affects career prospects and often influences the intention to migrate to more resourceful countries, especially among nurses and doctors [70,71]. On a lesser scale, this lack of progression is due to the lack of required professional qualifications and investment in updated professional training [63].

Another factor of tension between colleagues is the unfair promotion of a professional instead of another equally deserving. A promotion is a nuclear motivation factor, perceived as a way of growth, recognition, and advancement, and is hence interconnected with the self-esteem and personal and professional self-realization of workers [72].

### 4.3. Work Conditions

Work conditions are nuclear motivational factors, encompassing the physical space and physical resources that are fundamental to the activities conducted [5,42,45,57,59,60,64,73,74,75,76,77,78,79]. These are particularly impactful in rural areas.

A lack of electricity, necessary medicine and medical equipment, hospital materials (e.g., protection gloves, masks), and resources and degraded physical conditions of health units are the most commonly reported factors related to poor work conditions. The lack of essential goods, such as water, soap, and gloves, is described as a common reality in African countries [80]. In rural areas with few resources, the lack of supplies is often accompanied by poor equipment and infrastructure, which affects the work conditions and the workforce well-being (for example, the storage and preservation of food and water in extreme climacteric conditions) [81].

### 4.4. Remuneration

The most important motivational factor is undoubtedly the remuneration of health professionals. All studies mention this motivational factor as being detrimental to motivation as remuneration is perceived as lower than necessary or what would be fair. Thus, it is common for these professionals to work in both public and private sectors as a way to earn higher incomes. Article [34] states that the salary should be proportional to the work hours. Articles [60,62,72,73,82,83] emphasize the higher remuneration in the private sector and its impact on health professionals’ preference to work there, except for article [26], referring to the geographical context of India. At last, article [64] concluded that higher salaries are not associated with an increase in motivation, and that relationships among peers and with patients constitute the main motivational factor. Bratton et al., (2010) [84] also state that financial incentives should entail a particular group of rewards and not an isolated predictive factor. Furthermore, higher salaries should be accompanied by other rewards, such as more personal and professional stability, stronger peer relations, better work conditions, and work recognition.

### 4.5. Recognition, Self-Realization, and Responsibility

The articles [54,55,75,77,78,85,86,87,88,89] identify some intrinsic factors connected with higher levels of motivation. To be recognized and appreciated by supervisors, colleagues, and patients is a major reward attuned to a sense of value and contribution. The lack thereof is associated with less satisfaction and a worse work environment. Research on self-realization, work values, workplace commitment, work satisfaction, and performance have attracted ample research in recent decades [88], especially due to the relation between these key variables and engagement and work motivation [89,90,91].

### 4.6. Life-Long Learning and Professional Development

According to some studies (e.g., [55,59,60,66,78,92,93,94]), training programs and life-long learning are especially valued because they are perceived as important for the career progression of health professionals. The progress in their capacity and knowledgeability that may allow them to provide higher quality treatment enhances the motivational indexes, self-esteem, and strengthens the feeling of expected recognition by supervisors and patients. Health professionals describe the importance of ongoing training and updates with other professionals and physicians. In rural areas in developing countries, the lack of education training and qualification available to health professionals is highlighted.

## 5. Summary of Main Findings

This sample, retrieved from three databases, reflects the vitality of this field of inquiry, including a considerable number of articles with an increase of more than 12% per year. This is suggestive of a timely focus on health professionals’ motivation from the perspective of public administration and health management. Among the analyzed articles, four are conceptual and draw on document analysis and secondary data analysis. However, only a small portion draws on theories of motivation or more solid conceptual bases. Eighty-five articles (95.5%) analyze empirical data collected, quantitative methodology being the most frequent approach.

The collaboration among authors, besides highlighting the salience of the Anglo-Saxon-based scientific production, speaks favorably about the internationalization of this research, mostly on the following topics: motivation and public sector choice, the quality of health services and human resources, and health policy management. The analysis of co-citations shows stronger cross-country and continental links in three main research areas: PSM and organization management, motivation and organizational behavior, and policies for health motivation.

The focus on the field of health and its professionals derives from job requirements, financial and non-financial incentives, career opportunities, and continuous investment in education to maintain the quality and equity of their work [22].

Overall, Perry is the most relevant author, being connected to most of the publications given his role in developing and disseminating the concept of PSM, of which the scale of 24 items is one of the outputs [1]. Still, the majority of the researchers developed their own instrument combining items of several scales, of which the most common is Perry’s (1996). For instance, a transcultural adaptation of this scale was developed and applied by Kim et al. (2013), with an update of the four dimensions to attraction to public service, commitment to public values, compassion, and self-sacrifice. However, many of the scales employed do not measure motivation per se (as the PSM scale) but other factors that impact motivation at work. Examples include Challenge and Hindrance-related Self-Reported Stress—C-HSS [36], the Presenteeism Scale [37], and the Job Performance Scale [51]. There is, however, a wide dispersion of measurements and adaptations that impair a more systematic and robust comparison of factors and contexts.

In the analyzed studies, interestingly, the African continent is the most represented one, which can result from the scarcity of health professionals and material resources [25]. It is followed by Asia and Europe, with emphasis on the United Kingdom and the lack of human capital in the British health system [95].

More than half the studies draw on the public sector of health (n = 56), with 31 comparing both and two circumscribed to the private health sector. Although such results may be expected, the fact that the clear sectoral divide (public vs. private) has been undermined in recent decades (mainly due to the implementation of the NPM principles in the public sector, resulting in a broader definition of public service to include all forms of occupation that serve the community and not just those employed by the government directly, could reveal a different pattern of research, i.e., with more focus on the private sector instead. As far as PSM is concerned, although originally associated with public institutions and organizations, it has been argued that it applies to the private sector, as well, when it targets public interest.

Despite the variations according to geographical contexts and specific organizational cultures, there are common denominators affecting health employees’ motivation and work engagement that intertwine other-oriented and self-oriented motives. The more common elements refer to remuneration, available resources and working conditions, absenteeism, and frequency of contact and interaction with patients. All of the above adopt a more individualistic perspective of the health worker in relation to his/her professional work/context specificities and less of an organization-based viewpoint related to career development in the institution or management/leadership approaches.

Partially explained by the sample composition, these main motivational elements are reported in a more negative light (e.g., low salaries, lack of resources, and absenteeism). On the other hand, the recognition and valorization of their work by supervisors and patients is consensually seen as relevant, even more by health workers from less resourceful settings, as is the case in rural areas and developing countries.

The structural and chronic limitations that characterize those contexts may explain that, instead of physical and material incentives and conditions beyond personal control, stronger individual-based determinants are privileged, as symbolic and identity-based notions of contribution, respect, and recognition, altogether reflecting the inherent sense of value of health care provision.

Being consensually regarded as important factors in the motivation of health workers, health and public administration institutions should prioritize these criteria through direct and indirect mechanisms, assuring health workers that their efforts and contributions to society are clearly valued and recognized.

## 6. Final Remarks

Research on Public Service Motivation (PSM) started with an exploratory conceptual approach three decades ago [1,45,96]. With the evolution of the research stream, PSM gradually anchored in organizational management and policies. However, overall, it seems that the research on PSM in the field of health and human resources is gauging more attention in recent years, as shown by the increasing number of publications in diverse geographical realities. Focused on health professionals’ motivation, this study contributes a timely and useful systematization during the current challenging times for health institutions and their human resources. Its contribution is threefold: (i) the analysis of the scientific production meta data informs an integrated “shadow history” of this topic; (ii) the identification of the most productive authors and countries/regions provides well-founded insights into their cooperation networks and dynamics; and (iii) the characterization of the most relevant themes, topics, determining factors of motivation, and cultural patterns indicates future research avenues.

As with other methods, this systematic literature review presents limitations that should be considered in further studies. First, the selected documents only include those written in English, leaving aside (few) documents referring to more specific or endogenous contexts that can enrich further analyses. Second, only articles were considered, overlooking potential interesting contributions from other types of sources. Third, the geo-cultural heterogeneity of the sample impairs a more robust comparison between the interaction of motivational factors with specific cultural contexts and respective health policies and work conditions.

The lack of more cohesive theoretical and methodological approaches to address health professionals’ motivation, as shown in this study, reflects a somewhat loose and fragmented approach to this issue. The development of an overarching conceptual approach of motivation of health professionals, including a specific measure validated and adapted to specific geo-cultural contexts, would significantly benefit this line of research. A systematic comparison within countries with similar geo-cultural contexts (for example, samples from WEIRD and developing contexts) could contribute to identify the moderation role of specific healthcare contexts and policies in health workers’ motivation. It may also provide useful insight on how motivation is modeled by specific cultural context expectations and socio-economic conditions, strengthening the need to approach it from a socio-psychological perspective. Future research would also benefit from a transcultural framework since, as evident in the current pandemic, there are many common denominators motivating and shaping health care workers’ commitment to their professions.

## Figures and Tables

**Table 1:** Study quality assessment questions.

#	Study Quality Assessment Questions
Q1	Are the objectives of the study clearly identified?
Q2	Is the context of the study clearly stated?
Q3	Do the research methods support the aims of the study?
Q4	Does the study have a comprehensive description of the instruments/scales employed?
Q5	Is there a clear statement of the findings?
Q6	Are the limitations of the study discussed extensively and explicitly?

**Table 2:** Most relevant authors per number of documents.

Author	N	Institution	Research Area	Citations (Sum of Articles)	H-Index (Scopus)
Andersen, Lotte Bogh	3	Arizona State University	Political Science	98	22
Bellé, Nicola	2	Scuola Superiore Sant’Anna	Management	278	14
Blaauw, Duane	2	Univ. of the Witwatersrand Johannesburg	Health Policy	97	22
Alhassan, Robert Kaba	2	University of Health and Allied Sciences	Public Health	85	10
George, Asha S.	2	University of the Western Cape	Public Health	59	30
Bhatnagar, Aarushi	2	J.H. Bloomberg School of Public Health	International Health	59	10
Ferrinho, Paulo	2	IMHT—University of Lisbon	Human Resources for Health	52	18
Deng, Jianwei	2	Beijing Institute of Technology	Public Management	17	8
Belalia, Abdelmounin	2	National School of Public Health	Health Management	14	4
Belrhiti, Zakaria	2	National School of Public Health	Health Management	14	5

**Table 3:** Journals with three or more articles published.

Journal	Number of Articles	Subject Area and Category	Quartile (2020)
*Human Resources for Health*	15	Public Health, Environmental and Occupational Health	Q1
*Health Policy and Planning*	5	Health Policy	Q1
*BMC Health Services Research*	4	Health Policy	Q1
*International Journal of Public Administration*	4	Public Administration	Q2
*Global Health Action*	3	Health Policy	Q1
*International Public Management Journal*	3	Public Administration	Q1
*Social Science and Medicine*	3	Health (Social Science)	Q1

**Table 4:** Geographical distribution.

Country (ISO Code)	F	%	Continent	F	%
IN	7	7.87	Europe	22	24.44
DK, PK	6	6.74	North America	3	3.33
GH	5	5.62	Asia	24	26.67
CN, IT, TZ	4	4.49	Africa	36	40.00
NG, UK	3	3.37	South America	2	2.22
ZA, DE, ET, US, NL, MY, MW, MA, KN, ZM	2	2.25	Oceania	1	1.11
AO-GN-MZ-ST; AU; BD; BR; CV-GN-MZ; KH, CL, EG, UK-US; IL, NP, PL, PT, RO, RW, SE, TH, TP, UG	1	1.12	Not reported	2	2.22
Not reported	2	2.25			
Total	89	100	Total	90 ^1^	100

^1^ One article presents a comparative approach between North America and Europe.

**Table 5:** Scientific production and collaboration between countries.

Country	Nº Articles	SCP	MCP	MCP_Ratio
United Kingdom	8	6	2	0.25
USA	8	6	2	0.25
Denmark	5	5	0	0.00
South Africa	5	5	0	0.00
China	3	3	0	0.00
Ghana	3	2	1	0.33
India	3	3	0	0.00
Italy	3	3	0	0.00
The Netherlands	3	3	0	0.00
Germany	2	2	0	0.00

Country: affiliation country of corresponding author; Nº Articles: number of articles per affiliation country of the corresponding author; SCP: publication from one country; MCP: publications from several countries. Source: “Bibliometrix”.

**Table 6:** Analysis of co-citations—authors by clusters (“Bibliometrix”).

Cluster	Designation	Number of Authors	Main Authors	Focus of Work	Links (n)	Total Link Strength (n)
Red	PSM and Organization Management	17	Perry (University of Indiana)	PSM scale	41	2213
			Wright (Geórgia University)	Organizational behavior	40	1310
			Andersen (Aarhus University)	PSM and factors affecting professional performance	41	961
Blue	Motivation and Organizational Behavior	8	Brewe (Georgia University)	Public management and organizational behavior	40	1594
			Vandenabeele (Utrecht University)	Human resources motivation	40	1546
			Kim (Seoul University)	PSM, organizational behavior, and personnel management	36	1426
Green	Policies for Health Motivation	17	Franco (University Research Co)	Motivation of health professionals and sector reforms	37	312
			George (Western Cap University)	Motivation of health professionals	34	231
			Dieleman (Amsterdam’s Vrije Universiteit)	Health human resources policies and motivation determinants	34	196

Visualization in VOSviewer. Minimum number of documents per author: 2.42 items, 3 clusters, and 734 links.

**Table 7:** Number of publications by main types of health care provision.

Organizations	F	%
Health Units (various levels)	31	34.83
Hospitals	29	32.58
Primary Health Care	12	13.48
Universities	11	12.36
Third-Sector Entities	2	2.25
Pharmacies	2	2.25
Primary Health Care/Hospitals	1	1.12
Hospitals/Universities	1	1.12

**Table 8:** Type of professionals present in the articles.

Professionals	F
Physician	38
Nurses	29
Hospital Administrators/Managers	16
Health Professionals (no area specification)	14
Technicians	12
Health Students	11
Pharmacists	3
Dentists	1

**Table 9:** Main theories addressed in the analyzed studies.

Type of Theory of Motivation	Main Theories	Freq.
Needs-based	Maslow Theory	4
Needs-based	Self-Determination Theory—Deci and Ryan	3
Needs-based	McClelland Theory	3
Needs-based	Herzberg’s Two-Factor Theory	3
Task-based	Job Characteristics Model—Hackman and Oldham	3

**Table 10:** Brief description of the main motivation theories.

Motivation Theory	Brief Description
Maslow’s Hierarchy of Needs (Maslow, 1943)	A person is driven by achieving their necessities, from basic physiological factors, followed by security needs, safety, social interactions, self-esteem, and self-realization.
Job Characteristics Model (Hackman and Oldham, 1976)	There are five important work characteristics—skills, identity of the task, meaning of the task, feedback, and autonomy—which are essentially intrinsic motivational factors.
Self-Determination Theory (Deci and Ryan, 1980)	Explains the effects of the external consequences on intrinsic motivation. As such, external factors such as remuneration may contribute to question intrinsic behavior.
McClelland Theory (McClelland, 1960)	There are three main motivational drives in work contexts: realization, affiliation, and power. The first entails a need to achieve and show one’s own competences (self-realization). The second is an urge to be accepted and acknowledged by peers and others, and the third is the need to control their own work and the work of others.
Herzberg Theory (Herzberg, 1959)	Classifies the necessities in two categories: hygiene factors (or extrinsic) and motivational factors (or intrinsic). The motivational principles of this theory are achievement and recognition and the extrinsic remuneration and work safety.

**Table 11:** Instruments of data collection.

Methodological Method	Freq.	%
Questionnaire	42	47.19
Interviews	22	24.72
Interviews/Focus Group	6	6.74
Questionnaire/Interviews	6	6.74
Secondary Analysis of Primary Sources	4	4.49
Direct Observation	4	4.49
Questionnaire/Interviews/Focus Group	3	3.37
Questionnaire/Focus Group	1	1.12
Questionnaire/Direct Observation	1	1.12

**Table 12:** Extrinsic motivation factors.

Main Factors	F
Low Salaries	47
Lack of Resources/Poor Working Conditions	23
Career Development	11
High Workload	9
Workplace	8
Balance Between Work and Family Life	2
Distance to the Workplace	2
Performance Evaluation	1

**Table 13:** Intrinsic motivation factors.

Main Factors	F
Absenteeism	22
Frequency of Contact With Patients/Altruism	21
Job Security	16
Recognition of Work	11
More Autonomy	7
Provision of Care	3
Religion	1

## Data Availability

The review used existing research data.

## Appendix A

**Authors**	**Year**	**Remuneration**	**Work Environment/** **Work Conditions**	**Life-Long Learning/** **Professional Development**	**High Workload**	**Absenteeism**	**Altruism**	**Job Security**	**Recognition of Work**	**Responsibility/** **Autonomy**
Jiang et al. [97]	2021					x				
Stefurak et al. [98]	2020	x								
Cantarelli et al. [85]	2020	x			x		x			x
Ebenso et al. [99]	2020	x	x							
Jensen et al. [100]	2020	x			x		x	x	x	
Belrhiti et al. [86]	2020		x							x
Androniceanu et al. [73]	2020	x	x		x					
Abdelmotaleb et al. [101]	2020			x			x			
van Loon et al. [102]	2020						x		x	
Belrhiti et al. [86]	2019	x			x			x	x	
Deng et al. [103]	2019					x				
Deng et al. [104]	2019	x		x						
Adams et al. [105]	2019	x					x			
Prust et al. [75]	2019	x	x	x						
Chang et al. [63]	2019	x		x	x					
Borghi, et al. [106]	2018				x					x
Zweigenthal et al. [107]	2018		x				x			
Aguilera et al. [108]	2017						x			
Abera et al. [109]	2017	x	x			x				
Masood et al. [110]	2017									x
Levitats et al. [111]	2017		x							
Millar et al. [112]	2017	x		x	x		x			
Sato et al. [75]	2017	x	x							x
Korlén et al. [76]	2017		x	x			x			
Bhatnagar et al. [42]	2017	x	x						x	
Seth [113]	2017	x						x		
Mazahir et al. [77]	2017		x						x	
Hou et al. [114]	2016	x		x			x			
Russo et al. [82]	2016	x								
Bhatnagar and George [115]	2016		x				x		x	
Shah et al. [116]	2016	x	x	x	x			x		
Alhassan et al. [64]	2016	x	x	x			x			
Namakula et al. [117]	2016	x						x		
Bansal and Malhotra [71]	2016	x	x	x						
Van Loon [118]	2016		x						x	x
Kadam et al. [119]	2016	x		x						
Aninanya et al. [87]	2016	x					x		x	
Khim [55]	2016	x	x	x					x	
Mir et al. [61]	2015	x	x							
Hotchkiss et al. [53]	2015	x	x					x	x	
Gasiorowski et al. [56]	2015		x	x			x	x		
Ashmore and Gilson [120]	2015	x								
Shibata et al. [92]	2015	x		x						
Bellé [121]	2015	x								
Schmiedeknecht et al. [122]	2015	x	x		x		x	x		
Russo et al. [123]	2014	x			x					x
Bonenberger et al. [65]	2014	x	x	x						
Ojakaa et al. [57]	2014	x	x	x				x		
Hennig-Schmidt and Wiesen [124]	2014	x					x			
Purohit et al. [66]	2014	x	x	x			x	x	x	
Lagarde and Blaauw [125]	2014						x			
Krogsgaard et al. [126]	2014								x	
Schott and Pronk [127]	2014	x		x						x
Diwan et al. [128]	2013	x					x	x		
Alhassan et al. [129]	2013	x	x				x			
Kolstad et al. [130]	2013						x			
Ghimire et al. [57]	2013	x	x	x	x			x		
George et al. [131]	2013	x	x	x						
Mutale et al. [93]	2013	x	x	x	x					
Dos Santos et al. [132]	2013			x						
Bellé [133]	2013							x		
van Loon et al. [134]	2013						x			
Chew et al. [67]	2013		x				x			
Mariappan [135]	2013	x	x	x	x					
Ashmore [68]	2013				x					x
Andersen et al. [136]	2013	x	x	x	x			x		
Songstad et al. [137]	2012	x	x	x	x					
Waitzkin H. et al. [69]	2012	x	x					x		
Kjeldsen [138]	2012						x			
Georgellis et al. [139]	2011						x			
Aysen and Parumasur [140]	2011	x					x		x	
Halepota and Shah [95]	2011	x	x	x						
Leonard and Masatu [141]	2010	x	x	x	x					
Peters et al. [59]	2010	x	x	x						
Malik et al. [142]	2010	x	x	x	x		x	x		
Serneels et al. [143]	2010	x								
Le Julian [144]	2010									x
Mbindyo et al. [67]	2009	x	x	x	x		x	x	x	
Andersen [145]	2009	x								
Mangham and Hanson [146]	2008	x	x							
Modipa and Dambisya [147]	2008						x			
Dambisya et al. [79]	2007	x	x	x			x			
Agyepong et al. [148]	2004	x	x	x	x					
Humphrey and Russell [62]	2004	x	x						x	
Franco et al. [89]	2002	x	x				x		x	
Lee-Ross [149]	2002				x					x
Pipan [150]	2000									x
Smith [94]	1999	x	x	x						
Ferrinho et al. [83]	1998	x						x		
